# Authenticity Detection of Black Rice by Near-Infrared Spectroscopy and Support Vector Data Description

**DOI:** 10.1155/2018/8032831

**Published:** 2018-07-09

**Authors:** Hui Chen, Chao Tan, Zan Lin

**Affiliations:** ^1^Key Lab of Process Analysis and Control of Sichuan Universities, Yibin University, Yibin, Sichuan 644000, China; ^2^Hospital, Yibin University, Yibin, Sichuan 644000, China; ^3^The First Affiliated Hospital, Chongqing Medical University, Chongqing 400016, China

## Abstract

Black rice is an important rice species in Southeast Asia. It is a common phenomenon to pass low-priced black rice off as high-priced ones for economic benefit, especially in some remote towns. There is increasing need for the development of fast, easy-to-use, and low-cost analytical methods for authenticity detection. The feasibility to utilize near-infrared (NIR) spectroscopy and support vector data description (SVDD) for such a goal is explored. Principal component analysis (PCA) is used for exploratory analysis and feature extraction. Another two data description methods, i.e., k-nearest neighbor data description (KNNDD) and GAUSS method, are used as the reference. A total of 142 samples from three brands were collected for spectral analysis. Each time, the samples of a brand serve as the target class whereas other samples serve as the outlier class. Based on both the first two principal components (PCs) and original variables, three types of data descriptions were constructed. On average, the optimized SVDD model achieves acceptable performance, i.e., a specificity of 100% and a sensitivity of 94.2% on the independent test set with tight boundary. It indicates that SVDD combined with NIR is feasible and effective for authenticity detection of black rice.

## 1. Introduction

Black rice is an economically important special rice species and has been consumed for a long time in Southeast Asia including China [1–3]. Many researches have showed that black rice has considerably strong free-radical scavenging and antioxidation effects, as well as other biological effects of its extracts such as antimutagenic and anticarcinogenic [4, 5]. Black rice quality in terms of nutrition is also valuable for its protein content and the balance of essential amino acids. In fact, black rice is also a mixture of various carbohydrates. There exist varying amounts of nutrient in different kinds of black rice because of genetic and environmental factors. In market, there exist many brands of black rice. The quality and price of them vary greatly and renowned brands have higher price. However, illegal tradesman often passes low-priced black rice off as high-priced ones for economic benefit, especially in some remote towns.

How to discriminate different types of black rice is interesting. Up to now, it is mainly dependent on human senses. More objective and novel methods are maybe based on complex instruments such as high performance liquid chromatography or mass spectroscopy (MS) [6]. In recent years, molecular spectroscopy has drawn more attention and proved to be a powerful tool for authenticity detection [7–9]. In particular, near-infrared (NIR) spectroscopy becomes the most widely used technique in various fields including cigarettes [10], food [11], textile [12], medicine [13], and drug [14]. It is capable of rapidly obtaining a vector/matrix signal of a complex sample and therefore provides the chance of executing a in-depth qualitative or quantitative analysis. Detection of food authenticity is a important task in food analysis and aims to answer the question on which class a particular sample belongs to by its spectral signal. Often, it can be realized by comparing spectra of a specimen to be identified with spectra of “known” or “standard.” As for NIR spectroscopy, however, spectral signals for complex food systems are characterized by peak overlapping and poor resolution. So, an appropriate chemometric model is indispensable for a NIR-based application.

For the perspective of modeling, chemometrics involving qualitative tasks can be divided into two categories: classification and one-class classification, i.e., data description [15]. Classification is vey often considered as a synonym of discriminant analysis methods since they assign a new sample to one of a set of predefined classes. The corresponding classifier is trained on a training set. Data description differs in one essential aspect from the conventional classification since it is assumed that only information on a single class is available. Data description problems are common in the real world where positive objects are widely available but negative ones are maybe hard, expensive, or even impossible to gather [16]. In the literature, three main approaches can be distinguished: the density estimation, the boundary methods, and the reconstruction methods [17]. General demand of any authentication problem is that a genuine class, i.e., a target class, must be known [18, 19]. The target class is always unique for a specific authentication problem. Any other objects, or classes of objects, that are not members of the target class are considered as outliers. This also means that just samples of the target class can be utilized and that no information on the other classes is present. For data description, the boundary surrounding the target class has to be estimated from available data, such that it accepts as much of the target samples as possible and minimizes the error of accepting outlier. Up to now, much effort has been expended to develop classification algorithms, and the concept of data description is also of interest and noticeable [20–23], especially in the cases where it is impossible to meaningfully define all of the classes and obtain fully representative samples. In food authenticity, the interest is focused on a single target class so as to verify compliance of samples with the features of that class, and a data description approach should be adopted to build an enclosed boundary around the target class.

The present work focuses on exploring the feasibility to utilize near-infrared (NIR) spectroscopy and support vector data description (SVDD) for authenticity diction of black rice. Principal component analysis (PCA) is used for exploratory analysis and feature extraction. Another two data description methods, i.e., k-nearest neighbor data description (KNNDD) and GAUSS method, are used as the reference. A total of 142 samples from three brands were collected for spectral analysis. All spectra were preprocessed beforehand by standard normal transformation (SNV). Each time, the samples of a brand serve as the target class whereas other samples serve as the outlier class. Based on both the first two principal components (PCs) and original variables, three types of data descriptions were constructed. On average, the optimized SVDD model achieves acceptable performance, i.e., a specificity of 100% and a sensitivity of 94.2% on the independent test set with tight boundary. The effect of training set size and the parameter of kernel width have also been discussed. It indicates that SVDD combined with NIR is feasible and effective for authenticity detection of black rice.

## 2. Theory and Methods

Many methods have been developed to solve the one-class or data description problem and they can be divided into three main categories: density, boundary, and reconstruction methods. Here, three algorithms, i.e., support vector data description, Nearest Neighbor Method, and Gaussian Method, are introduced and used for experiments, among which the first two are boundary methods and the last one belongs to density method.

### 2.1. Support Vector Data Description (SVDD)

SVDD is a novel algorithm for one-class classification problems, which has been proposed by Tax [15], inspired by the idea of the support vector machines. It focuses on finding a minimum hypersphere around the target class. The hypersphere can be used to decide whether new objects are targets or outliers. Such a sphere is characterized only by center **a** and radius *R*. When seeking sphere, it needs to minimize the volume of the sphere by minimizing *R*^2^ and demand that the sphere covers as many training samples as possible. Given the training set {**x**_*i*_, *i* = 1,2,…, *N*}, the task in SVDD is to minimize error function:

(1) min⁡ LR,a,ζi=R2+C∑iζi

(2) s.t. xi−a≤R2+ζi,ζi≥0  ∀i

where **a** and *R* are the center and the radius of the hypersphere, respectively; *C* is the penalty factor which regulates the hyperspherical volume and error, i.e., the number of target objects rejected; *ζ*_*i*_ is a slack variable for allowable error limitation. Almost all objects are within the sphere. This optimization problem can be solved by Lagrange multiplier method [24].

Because the target class is not spherically distributed in most cases, some traditional decision rules may not work well. To make a more effective and flexible decision, the original data can be implicitly transformed to a higher dimension by the so-called kernel function *K*(**x**_*i*_, **x**_*j*_). Several kernel functions including linear, polynomial, Gaussian, radial basis function (RBF) are available [25, 26]. In this work, the RBF kernel, the most commonly used kernel in machine learning, was used. The form of RBF kernel is

(3) $$\begin{array}{c} K\left(\;x_{i},x_{j}\;\right)=\exp\left(\;-\frac{{\left\|x_{i}-x_{j}\right\|}^{2}}{\sigma^{2}}\;\right) \end{array}$$

where *σ* is a key parameter for controlling the boundary tightness.

### 2.2. Nearest Neighbor Method

The most straightforward and simplest method to obtain a one-class model is to estimate the density of the training set. Unfortunately, it often requires a large number of samples to avoid the curse of dimensionality. Instead of estimating whole probability densities, an indication of the resemblance can also be acquired by comparing distances. Nearest neighbor method can be derived from a local density estimation [27]. It avoids the explicit density estimation by only using distances to the first nearest neighbor. In the process of density estimation, a cell, often an hypersphere in d-dimension space, is centered around the test object **z**. The cell volume is grown until it contains *k* objects from the training set. The local density can be estimated by

(4) $$\begin{array}{c} p_{NN}\left(\;z\;\right)=\frac{k/N}{V_{k}\left\|z-NN_{k}^{tr}\left(z\right)\right\|} \end{array}$$

where *NN*_*k*_^*tr*^(**z**) and *V*_*k*_ are the *k* nearest neighbors of **z** in the training set and the volume of the cell containing this object. Later, we will use KNNDD to denote this method.

For an unknown test object **z**, the distance from it to its nearest neighbor in the training set NN^*tr*^(**z**) is compared with the distance from NN^*tr*^(**z**) to its nearest neighbor. The test object **z** can be accepted when its local density is larger or equal to the density of the nearest neighbor. It seems to be very useful for distributions characterized by fast decaying probabilities. Obviously, the method can easily be generalized to a larger number of neighbors k. That is, instead of taking the first nearest neighbor into account, the *k*th neighbor should be considered.

### 2.3. Gaussian Method

When a proper probability model is assumed and the sample size is sufficient, density method is advantageous for one-class problem. With the optimization of the threshold, a minimum volume can be automatically found for the given probability density. When only a little amount of samples is available, the simplest model is the unimodal Gaussian/Normal distribution. It fits a probability density model as follows:

(5) $$\begin{array}{c} p_{N}\left(\;x\;\right)\frac{1}{{\left(2\pi \right)}^{d/2}{\left|\Sigma \right|}^{0.5}}\exp\left\{\;-\frac{1}{2}{\left(\;x-\mu \;\right)}^{T}\Sigma^{-1}\left(\;x-\mu \;\right)\;\right\} \end{array}$$

where ***μ*** is the mean and Σ is the covariance matrix. Both should be estimated from the training set. For *d* dimensional data, the number of the parameters is

(6) $$\begin{array}{c} d+\frac{1}{2}d\left(\;d-1\;\right) \end{array}$$

The method imposes a strict unimodal and convex density model on the data. The main computational effort is maybe the inversion of the covariance matrix. In case of badly scaled data or data with singular directions, it is difficult to calculate the inverse of Σ and it can be approximated by the pseudoinverse Σ^+^ = Σ^*T*^(ΣΣ^*T*^)^−1^or by introducing regularization (adding a small constant *λ* to the diagonal, i.e., Σ′ = Σ + *λ ***I**). In the last case, the user needs to supply a parameter *λ*. This is also the only magic parameter that requires a user to provide.

Finally, a threshold on the probability density needs to be set for distinguishing between target and outlier data. Accepting 95% of the objects requires a threshold on the Mahanalobis distance

(7) $$\begin{array}{c} {\left(\;x-\mu \;\right)}^{T}\Sigma^{-1}\left(\;x-\mu \;\right) \end{array}$$

of

(8) $$\begin{array}{c} \theta_{N}={\left(\;\chi_{d}^{2}\;\right)}^{-1}\left(\;0.95\;\right) \end{array}$$

where (*χ*_*d*_^2^)^−1^is the inverse *χ*_*d*_^2^ with *d* degrees of freedom. This method is expected to work effectively only if the data is unimodal and convex. To obtain a more flexible density method, it can be extended to a mixture of Gaussians. Later, we will use GAUSS to denote this method.

## 3. Experimental

### 3.1. Sample Preparation

A total of 142 samples/bag of black rice of three brands were purchased from local supermarkets in China. They were from different supplier and let us mark them as A, B, and C brands. These samples were collected from three batches of A, two batches of B, and three batches of C but different packages. For A or C, forty-eight bags of rice were sampled, sixteen bags for each batch; For B, forty-six bags of rice were sampled, twenty-three bags for each batch. In total, the number of samples belonging to A, B, and C are 48, 46, and 48, respectively. The time it takes to collect the sample is about six months. The samples of each brand could serve as the target class whereas other samples acted as the outlier class. All samples were stored in the laboratory kept at 25°C for more than 7 days in order to achieve a temperature balance. To reduce the effect of environment, the NIR spectra of all samples were recorded on the same day.

### 3.2. Spectral Measurement and Preprocessing

Spectra of different samples collected on an Antaris II FT-NIR spectrometer (Thermo Scientific Co./USA) were equipped with an integrating sphere module, a rotating sample cup, and a InGaAs detector, as well as a tungsten lamp as the light source. The sample was poured into a standard sample cup with a 50 mm diameter and the height was controlled on about** 30 mm** for preventing light leak. An internal gold reference was used for automatic background collection. A specific sample cup spinner accessory for the integrating sphere sampling module that allows multipoint reflection measurements of heterogeneous solids such as powders, granules, and pellets, was used for obtaining NIR spectra of high quality. In this way, the final spectrum is the average of the spectra collected at different locations, which can reduce the effect of heterogeneity of solids to some extent.

The NIR spectrum was measured in the region of 10,000–4000 cm^−1^ with 32 scans at a resolution of 3.856 cm^−1^. Each spectrum contains 1557 data points. The experimental temperature and the related humidity were controlled around 25°C and 60%, respectively. Preprocessing of spectra is often of great importance if reasonable results need to be obtained whether it is concerned with qualitative or quantitative tasks. Several methods of preprocessing were attempted. In comparison with other preprocessing methods, standard normal transformation (SNV) achieved a satisfactory performance without the need of a reference spectrum and user decision for the computation. So, all spectra were preprocessed by SNV. The spectral measurement was controlled by the Result software [28]. DD toolbox was used for one-class classifier modeling [15]. All calculation was made on MATLAB 2015b for Windows.

## 4. Results and Discussions

### 4.1. NIR Spectral Analysis

**Figure 1:**
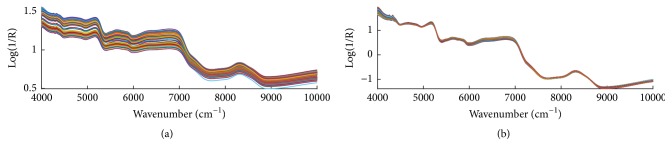
Original near-infrared (NIR) spectra (a) and all the preprocessed spectra (b) by standard normal transformation (SNV).

Figure 1 shows the NIR spectra and all the preprocessed spectra of black rice samples by SNV. Seen from Figure 1, the spectra of three types of black rice share very similar absorbance patterns in the range of 4000-11000 cm^−1^. They can hardly be distinguished just by naked eyes. General features of a NIR spectrum of solid samples include a multiplicative response to changes in particle size. SNV treatment autoscales each spectrum based on calculating the mean and standard deviation between the densities. It is also clear in Figure 1 that, by preprocessing, some additive and multiplicative effects have been removed.

It is well known that major components of black rice are complex molecules from the polymerization of monomers such as amino acids or carbohydrates. Each monomer exhibits specific chemical groups such as carboxylic and amine functions in amino acids. As each chemical group may absorb the infrared region light, it appears useful to clearly identify the characteristic NIR bands of these groups. Because NIR spectrum corresponds to molecular responses of the overtone and combination bands, for each fundamental absorption band, there exists several overtones with decreasing intensity corresponding to the increasing multiple or transition number. All the bands can form a myriad of combination bands with intensities increasing as frequency decreases. NIR band intensities are much weaker than their corresponding mid-infrared fundamentals by a factor of 10-100. In Figure 1, two strong bands at 5175 cm^−1^ and 6930 cm^−1^ result from the absorbance of water, among which the peaks around 5175 cm^−1^ are the combination of asymmetric stretching and bending vibration of H_2_O. The band of 8200-8600 cm^−1^ can be attributed to the second overtones of C-H stretching in various groups. The wider bands in 6100–7000 cm^−1^ are mainly caused by the overlapping of the first overtones of O-H and N-H stretching. The two peaks at 4266cm^−1^ and 4335cm^−1^, which can be attributed to C-H stretching and C-H deformation, are very stable and carry much useful information. However, accurate assignments of each peak were maybe difficult due to low resolution and baseline shift; therefore, it is necessary to resort to chemometric methods to extract the useful information from spectra for identification purposes.

Furthermore, one of the most interesting applications of NIR technique in the food analysis is total quality evaluation as it can provide fingerprint information of a sample. Different brands of black rice mean different balances/ratios of diverse chemical constituents and physicochemical properties, rather than simple amount of each constituent. NIR spectra contain rich information on chemical constituents and physicochemical properties. Although the quality of black rice is generally assessed by sensory evaluation, its taste is actually a function of chemical constituents such as protein, moisture, amylose, fatty acid, and minerals. Therefore, an overall evaluation is preferred based on NIR spectroscopy.

Principal component analysis (PCA), the most widespread multivariate tool, was used for an exploratory analysis and dimensional reduction. Unlike other applications, the main goal of the present work using PCA was to map the original data into its principal component score space (i.e., the first two), based on which the subsequent modeling was carried out. So, all samples were considered as a whole for PCA and mean-centering pretreatment. By computation, the first two PCs explain 79.4% and 18.4% of the total variances, respectively, and they may contain most of the useful information in the original spectra. Because of this, we decided to use the first two components as the input of subsequent data description methods.

### 4.2. Authenticity Detection by Data Description

**Figure 2:**
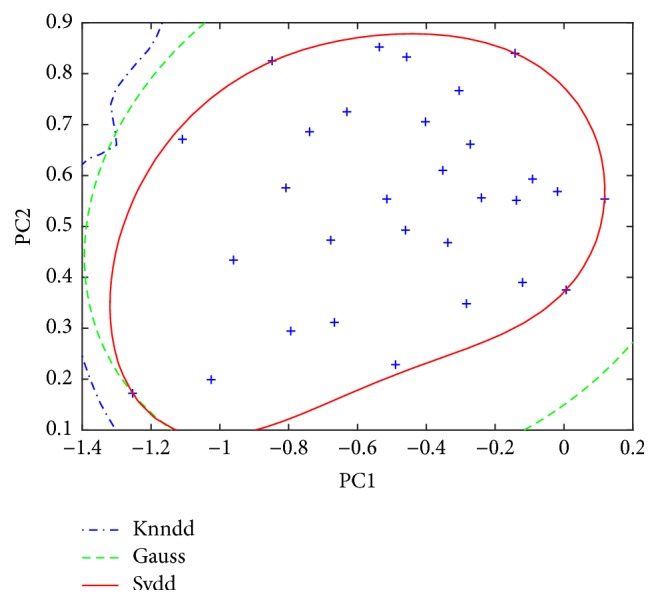
Data description boundary of class A on the first two-principal-component space based on the training set.

Given a dataset, in general, the selection of a representative training set upon which training the prediction model is performed is very important. For this purpose, in our work, the Kennard and Stone (KS) algorithm [29] was first used to rank all samples of each class in the dataset under consideration, thereby producing three sequences (A, B, and C). The KS algorithm consists of two main steps: taking the pair of samples between which the Euclidean distance of x-vectors (predictor) is largest, and then sequentially selecting a sample to maximize the Euclidean distances between x-vectors of already selected samples and the remaining samples. This process is repeated until all samples are picked out. The former samples are more representative than the latter one. When A class served as the target class, only the first thirty samples in A sequence were used as the training set for constructing data description. The remaining samples in A sequence and all samples in B and C sequences were used as the test set (the same partition of the sample set for the cases using B or C as the target class). Based on the first two PCs of the training set, three types of data descriptions mentioned above, i.e., SVDD, KNNDD, and GAUSS, were constructed. SVDD used the Gaussian kernel.  Figure 2 gives the optimized data description boundary of class A based on the training set. It seems that the boundary of SVDD is tightest. All the descriptions differ from conventional classification because they always obtain a closed boundary around one of the target classes. Unlike density methods such as GAUSS, SVDD does not require a strict representative sampling of the target class; a sampling containing extreme objects is also acceptable. This can be found explicitly in the error definition of SVDD, which minimizes the volume of the description plus the sum of slack variables for objects outside the description. A conventional classifier, on the contrary, distinguishes between two/multiple classes without focusing on any of the classes and aims to minimize the probability of overall error. It is expected to perform very poorly when just the target class is available or the dataset is relatively small. Food validation or authenticity detection is often the case.

**Figure 3:**
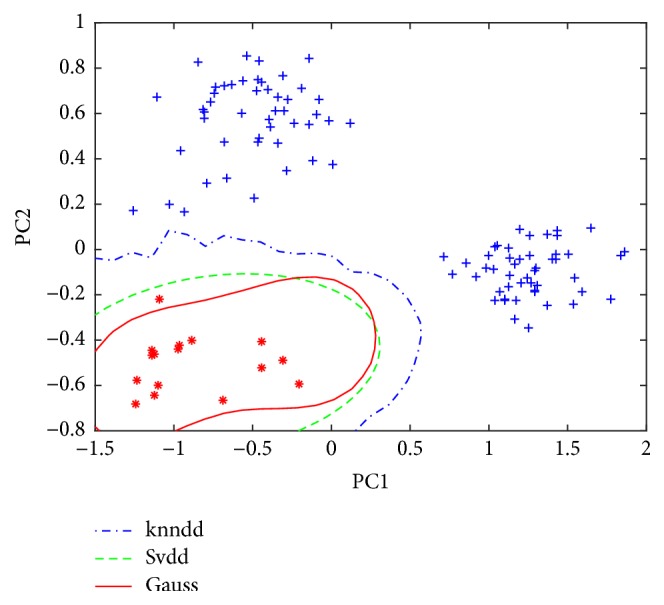
Application of the data description models of class A on the test set.

**Figure 4:**
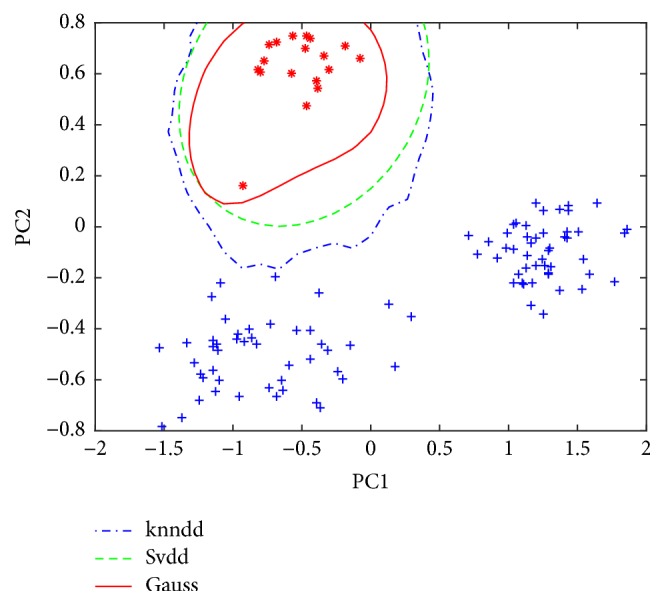
Application of the data description models of class B on the test set.

Figure 3 shows the application of the data description models of class A on the test set. Only one target sample was identified as outlier by SVDD. Even if the KNNDD and GAUSS correctly identified all samples, the false positive would increase when more test samples were used in the future. Similarly, Classes B and C were considered as the target class, and three corresponding data descriptions were constructed.  Figure 4 shows the application of the data description models of class B on the test sets. Now, all the models correctly identified the target samples and the corresponding outliers but the SVDD use the tightest boundary, maybe implying better generalization ability. Both KNNDD and GAUSS produce looser borders. It should be noted that each time the so-called “fake” black rice is actually simulated by the samples from nontarget class.

**Figure 5:**
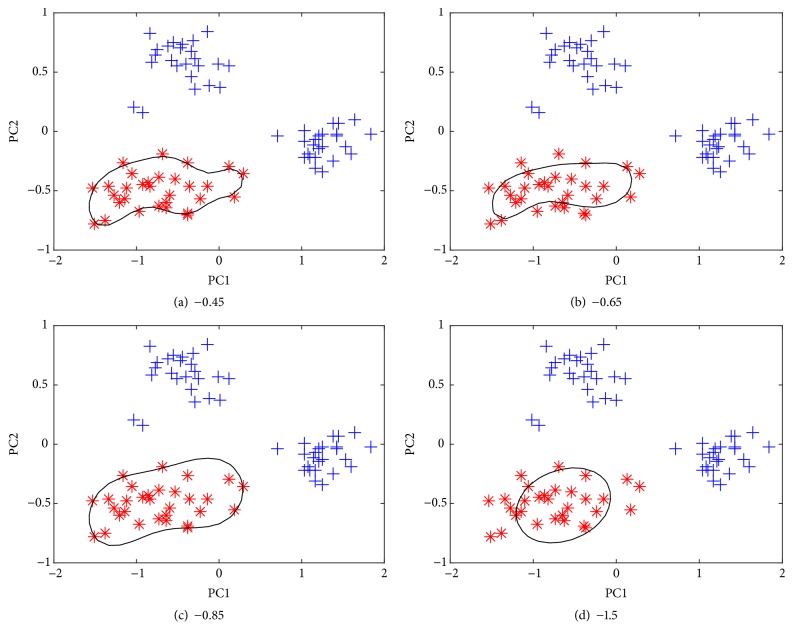
The influence of the kernel parameter on the boundary of support vector data description (SVDD) using class A as the target class (classification error on the target class is set as 0.1).

**Figure 6:**
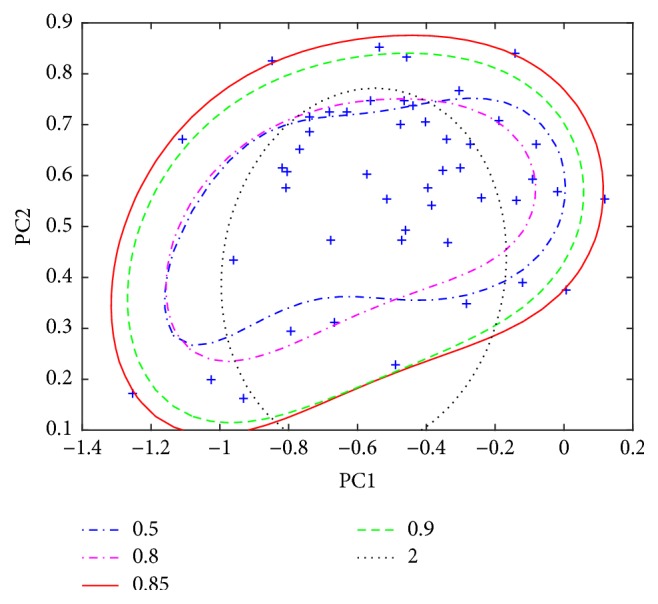
Ensemble of the influence of the kernel parameter on the boundary of support vector data description (SVDD) on the same plot.

The character of the SVDD heavily depends on the width parameter of the Gaussian kernel, which is very crucial as it can provide different prediction performance and leads to overfitting problem. Several previous studies have reported how to optimize SVDD [30]. The penalty term is sample rejection rate, i.e., the approximate proportion of samples misclassified in a training set. The other tunable parameter is kernel width. A large width can lead to a less complicated boundary and a relatively large width (compared to the maximum distance between samples in training set) could lead to a rigid hypersphere. In this work, based on the average nearest neighbor distance in the dataset, one can distinguish three types of cases: very small, very large, and intermediate values. By changing the value, the description ranges from Parzen density estimation, via a mixture of Gaussian to the rigid hypersphere, can be observed in Figure 5, which shows the influence of the kernel parameter on the boundary of SVDD when using class A as the target class. The boundary of SVDD seems to be sensitive to the kernel parameter. With the increase of the width, the boundary undergoes a complex change; it gradually achieves the optimum and then gets worse. For different cases, the number of support vectors is also different. In order to facilitate the comparison, Figure 6 gives a similar ensemble plot of the influence of the kernel parameter on the boundary. Based on the shape and the compactness of the edge of the description, the optimal width parameter is 0.85 for this case A. Such a boundary contains all the target samples, among which six samples are just on the edge, and the shape is also simple. Also, one important advantage of SVDD over some traditional methods is that the classifier does not require that the data follow a normal distribution. However, there exist some alternative procedures for optimizing kernel width such as cross-validation, bootstrapping, and the consistency evaluation of the classifier using only the error of the nontarget class [15, 30, 31].

Taking the first case as an example (A as the target class), instead of the PCs, the original spectral variables were used as the independent variables for constructing data descriptions. On the independent test set, all these models including SVDD, KNNDD, and GAUSS achieved a specificity of 100% (the ration of outliers that were rejected), while the corresponding sensitivity, i.e., the ratio of the target class that was accepted, is 100% for GUASS and 94.4% for both SVDD and KNNDD, despite whether PCs or original variables are used. It indicates that using PCs or original variables does not make substantial difference. However, using all features is likely to result in overfitting, while using PCs will likely reduce overfitting. Also, using PCs makes the computation to be faster and to be more convenient for visual purposes. When B or C is the target class, the corresponding specificity and sensitivity have also been summarized in Table 1. On average, the SVDD achieves best prediction, with the specificity of 100% and the sensitivity of 94.2%.

On the whole, the data description, especially SVDD, achieved an acceptable sensitivity and specificity for the so-called small-sample problem. Such a procedure is maybe potential tool for authenticity detection of various foods including black rice.

## 5. Conclusions

The work reveals that NIR spectroscopy combined with support vector data description is feasible and advantageous to implement authenticity detection of black rice. It can serve as an alternative to laborious, time-consuming, wet chemical methods and sensory analysis of human. However for obtaining more reliable results, more samples need to be collected, which remains our next work.

## Figures and Tables

**Table 1:** Summary of the performance of different models.

Target class	GAUSS		KNNDD		SVDD	
	SPE	SEN	SPE	SEN	SPE	SEN
A	100%	100%	100%	94.4%	100%	94.4%
B	96.8%	87.5%	96.8%%	93.8%	100%	93.8%
C	97.8%	88.9%	98.9%	88.9%	100%	94.4%
Average	98.2%	92.1%	98.5%	92.3%	100%	94.2%

*Note*. SPE and SEN denote the specificity and sensitivity, respectively.

## Data Availability

The spectra data used to support the findings of this study are available from the corresponding author upon request.
